# Cell type dependent stability and virulence of a recombinant SARS-CoV-2, and engineering of a propagation deficient RNA replicon to analyze virus RNA synthesis

**DOI:** 10.3389/fcimb.2023.1268227

**Published:** 2023-10-24

**Authors:** Li Wang, María Guzman, Diego Muñoz-Santos, Jose Manuel Honrubia, Jorge Ripoll-Gomez, Rafael Delgado, Isabel Sola, Luis Enjuanes, Sonia Zuñiga

**Affiliations:** ^1^ Department of Molecular and Cell Biology, National Center of Biotechnology (CNB-CSIC), Madrid, Spain; ^2^ Laboratory of Molecular Microbiology, Instituto de Investigación Hospital 12 de Octubre (Imas12), Madrid, Spain

**Keywords:** coronavirus, SARS-CoV-2, infectious cDNA, replicon, virulence, replication

## Abstract

Engineering of reverse genetics systems for newly emerged viruses allows viral genome manipulation, being an essential tool for the study of virus life cycle, virus-host interactions and pathogenesis, as well as for the development of effective antiviral strategies. Severe acute respiratory syndrome coronavirus 2 (SARS-CoV-2) is an emergent human coronavirus that has caused the coronavirus disease (COVID-19) pandemic. The engineering of a full-length infectious cDNA clone and a fluorescent replicon of SARS-CoV-2 Wuhan-Hu-1, using a bacterial artificial chromosome, is reported. Viral growth and genetic stability in eleven cell lines were analyzed, showing that both VeroE6 cells overexpressing transmembrane serin protease 2 (TMPRSS2) and human lung derived cells resulted in the optimization of a cell system to preserve SARS-CoV-2 genetic stability. The recombinant SARS-CoV-2 virus and a point mutant expressing the D614G spike protein variant were virulent in a mouse model. The RNA replicon was propagation-defective, allowing its use in BSL-2 conditions to analyze viral RNA synthesis. The SARS-CoV-2 reverse genetics systems developed constitute a useful tool for studying the molecular biology of the virus, the development of genetically defined vaccines and to establish systems for antiviral compounds screening.

## 1 Introduction

Severe acute respiratory syndrome coronavirus-2 (SARS-CoV-2) is a life-threatening human virus that emerged in December 2019 and has caused a global epidemic with more than 770 million cases and more than 6.9 million deaths worldwide (WHO, September 24^th^, 2023) (Zhou et al., 2020; Zhu et al., 2020). The emergence of SARS-CoV-2 has highlighted the need for flexible broad therapeutic strategies to fight currently circulating CoVs and to control future epidemics.

SARS-CoV-2 is classified within the subgenus *Sarbecovirus* of genus *Betacoronavirus* (Wong et al., 2019; Woo et al., 2023). The positive-sense single-stranded RNA genome of SARS-CoV-2 has approximately 30 Kb, and contains 12 open reading frames (ORFs) in the order 5’- ORF1a, ORF1b, S, 3, E, M, 6, 7a, 7b, 8, N, 9b -3’ which are expressed from a nested set of nine mRNAs (Kim et al., 2020; Finkel et al., 2021; Kim et al., 2021; Zou et al., 2021). Genus-specific genes 3, 6, 7a, 7b and 8 are non-essential for virus replication (Silvas et al., 2021; Liu et al., 2022; McGrath et al., 2022). These genes have been involved in the modulation of virus-host interaction when overexpressed in cells, affecting processes such as interferon (IFN) response or pro-inflammatory pathways, involved in virus pathogenesis (Li et al., 2020a; Miorin et al., 2020; Lowery et al., 2021; Martin-Sancho et al., 2021; Redondo et al., 2021; Wu et al., 2022). However, there is limited information on the role of genus-specific genes in the context of viral infection (Silvas et al., 2021; Kee et al., 2022; McGrath et al., 2022).

The use of reverse genetics systems allows selective and controlled modifications in viral genomes. This implies a great progress for the study of specific viral genes involved in virus-host interactions and pathogenesis as well as the development of novel antiviral strategies, including next generation vaccines or antivirals (Enjuanes et al., 2016). Our laboratory used bacterial artificial chromosomes (BACs) to engineer transmissible gastroenteritis virus (TGEV) infectious cDNA, being the first cDNA clone engineered for a CoV (Almazan et al., 2000). This approach has successfully been applied in our laboratory to engineer infectious cDNA clones for other CoVs, such as SARS-CoV, Middle East Respiratory Syndrome CoV (MERS-CoV), human HCoV-OC43 and feline infectious peritonitis virus (FIPV) (Almazan et al., 2006; St-Jean et al., 2006; Balint et al., 2012; Almazan et al., 2013).

Replicons are self-amplifying nucleic acids that contain all viral proteins and RNA signals required for viral RNA synthesis and, in some cases, reporter genes to facilitate the analyses. Furthermore, replicons may lack structural genes, or other genes non-essential for genome replication, and no infectious viral particles are produced, thereby enabling safe handling under BSL-2 conditions (Almazan et al., 2006; Almazan et al., 2014). These replicons are relevant to evaluate essential aspects of viral RNA synthesis or to establish antiviral testing systems, independently of viral entry, morphogenesis and egress processes (Kato and Hishiki, 2016; Fernandes et al., 2020; Hannemann, 2020; Khan et al., 2020; Kurhade et al., 2023).

Angiotensin converting enzyme 2 (ACE2) is the main cellular receptor for SARS-CoV-2 (Zhou et al., 2020) and the major factor for different cell species susceptibility to SARS-CoV-2 infection (Lam et al., 2020; Li et al., 2020b; Shang et al., 2020; Zhai et al., 2020; Liu et al., 2021; Li et al., 2023). The role of cell proteases has also been described for efficient virus entry into the cells, such as transmembrane serin protease 2 (TMPRSS2) (Hoffmann et al., 2020b; Letko et al., 2020; Matsuyama et al., 2020). A set of entry co-factors has also been described for SARS-CoV-2 infection of different cell types (Singh et al., 2020; Peng et al., 2021; Jackson et al., 2022). Adaptation to host cell proteases has also been described, leading in many cases to the loss of furin cleavage site, which was deleterious for viral entry in some cell lines (Peacock et al., 2021; Chaudhry et al., 2022). Cell susceptibility and optimum virus replication depends on viral entry and also on host cell factors, such as the antiviral response or the presence of proviral host factors. Many cell systems were described as suitable for SARS-CoV-2 infection studies based on the use of pseudotyped viruses, which do not reflect the complexity of virus-host interactions influencing SARS-CoV-2 replication (Hoffmann et al., 2020a; Hoffmann et al., 2020b; Jackson et al., 2022).

In this work, the engineering of an infectious cDNA and a reporter RNA replicon for SARS-CoV-2 are being described. These are useful tools to analyze virus stability in different cell lines, virus-host interactions and pathogenesis.

## 2 Materials and methods

### 2.1 Ethics statement

Experiments involving animals were performed in strict accordance with EU (2010/63/UE) and Spanish (RD 53/2013 and 32/2007) guidelines. All the protocols were approved by the on-site Ethical Committee (permit n°. PROEX 146.6/20). Infected mice were housed in a self-contained ventilated rack (Allentown, NJ).

### 2.2 Cell lines

Monkey Vero E6 (ATCC CRL-1586), hamster kidney (BHK-21, CCL-10) and human normal lung fibroblasts (MRC-5, CCL-171) cells were obtained from American Type Culture Collection. Vero E6 cells expressing human TMPRSS2 protease (VeroE6-TMPRSS2, JCRB1819) were obtained from Japanese Collection of Research Bioresources. Human liver-derived Huh-7 cells were kindly provided by R. Bartenschlager (University of Heidelberg, Germany). The bronchial epithelial cell line Calu-3 2B4 (Yoshikawa et al., 2010) was kindly provided by C.T. Tseng (University of Texas Medical Branch, USA). Human lung-derived A549 cells expressing human ACE2 (A549-ACE2) cells were kindly provided by R. Andino (University of California San Francisco, USA) and A549 cells expressing both human ACE2 and TMPRSS2 (A549-ACE2-TMPRSS2) were obtained from InvivoGen (San Diego, CA, USA). Human kidney-derived cells expressing human ACE2 (HEK293-ACE2) were obtained from Innoprot (Derio, Spain). Cells were cultured in Dulbecco’s Modified Eagle Medium (DMEM, Lonza) supplemented with 25 mM HEPES, 10% fetal bovine serum (FBS, HyClone), 2% glutamine, 1% non-essential amino acids (Sigma) and maintained at 37°C in a humidified atmosphere of 5% CO_2_. Calu-3 2B4 cells were grown in the medium described above supplemented with 20% FBS. Several cell lines required antibiotics for maintenance: 1 mg/ml of G418 for Huh7-ACE2, A549-ACE2 and VeroE6-TMPRSS2 cells; 80 µg/ml of hygromycin for HEK293-ACE2 cells; 0.5 µg/ml of puromycin and 300 µg/ml of hygromycin for A549-ACE2-TMPRSS2.

### 2.3 Viruses

Recombinant SARS-CoV-2 (rSARS-CoV-2) viruses were rescued from the corresponding infectious cDNAs. To obtain a SARS-CoV-2 clinical isolate as a control, six nasopharyngeal swabs from COVID-19 patients were kindly provided by R. Delgado (Hospital 12 de Octubre, Madrid, Spain). The swab samples were diluted in 500 µl of cell culture medium. Confluent Vero E6 cells, cultured as indicated above in a F12.5 flask, with medium supplemented with 100 U/ml penicillin, 100 mg/ml streptomycin, 50 mg/ml gentamicin and 0.5 mg/ml amphotericin B, were infected with half of the clinical sample. At 48 hpi, supernatants were collected and filtered through a 0.22 µm Millex-GS filter (Millipore) to eliminate potential bacterial and fungi contamination. Total RNA was extracted from half of the supernatant, using QIAamp Viral Mini kit (Qiagen) following the manufacturer’s instructions, and sent for next generation sequencing at Hospital 12 de Octubre (Madrid, Spain). Attending to the observed cytopathic effect and the obtained sequencing data, SARS-CoV-2-MAD6 was selected, which derived from a 69-year-old male patient clinical sample. The virus was plaque purified three times and viral stock was prepared following standard procedures set up in our laboratory (DeDiego et al., 2007). Virus titration was performed in Vero E6 cells, following standard procedures (DeDiego et al., 2007). All experiments with SARS-CoV-2 infectious viruses were performed in BSL-3 facilities at CNB-CSIC according to the guidelines set forth by the institution.

### 2.4 Generation of Huh-7 cells overexpressing human ACE2

Immunofluorescence analysis of human ACE2 expression in Huh-7 cells showed heterogeneous expression of this protein (data not shown). Then, two strategies were followed. First, Huh-7 cells were reverse transfected with 2 µg of plasmid pcDNA-hACE2, encoding human ACE2, kindly provided by M. Farzan (Harvard Medical School, USA) (Li et al., 2004), using LipofectamineTM 2000 (ThermoFisher Scientific) following the manufacturer’s recommendations. At 48 h post transfection (hpt) G418 (1 mg/ml) selection was applied for 10-12 days. The G418-containing growth medium was replaced every two days and cell toxicity was visually examined. Huh-7-ACE2 cells were then infected for subsequent analysis. The second strategy was to isolate independent cell clones from Huh-7 cells and select for high ACE2 expression levels. After several rounds of cell cloning by limiting dilution, seven independent ACE2-positive clones were selected by immunofluorescence. Huh-7 1D11 cell clone was finally selected based on high ACE2 expression levels and other cell characteristics, such as duplication time, morphology, etc.

### 2.5 Engineering of SARS-CoV-2 infectious cDNAs

To engineer a SARS-CoV-2 infectious cDNA (pBAC-SARSCoV-2^FL^), six DNA fragments were chemically synthesized and purchased from GenScript (New Jersey, USA) (**Table 1**). The full-length cDNA clone (pBAC-SARSCoV2^FL^) was assembled by sequential cloning of overlapping DNA fragments (F1-F6), covering the entire SARS-CoV-2 Wuhan-Hu-1 isolate (SARS-CoV-2-WH1) genome (GenBank MN908947), into the plasmid pBAC using the unique restriction sites selected shown in **Table 1**.

**Table 1:** DNA fragments for SARS-CoV-2 full-length cDNA clone assembly.

			RESTRICTION SITES		
N°	SIZE (bp)	VIRAL GENOME (nt) ^(a)^	5’ END	3’ END	CLONED INTO ^(b)^
1	957	1-346	*Asc*I	*Bsi*WI	pUC57-F1
2	6,401	347-6,747	*Bsi*WI	*Pme*I	pUC57-F2
3	7,209	6,748-13,956	*Pme*I	*Mlu*I	pCC1-4k-F3
4	6,130	13,957-20,086	*Mlu*I	*San*DI	pUC57-F4
5	5,227	20,087-25,313	*San*DI	*Bam*HI	pUC57-F5
6	4,612	25,314-29,870	*Bam*HI	*Rsr*II	pUC57-F6

^(a)^ Viral genome nucleotides, in agreement with GenBank MN908947.

^(b)^ Fragments F1 to F6 in agreement with **Figure 1A**. Plasmids were obtained from GenScript.

The plasmid pBAC-SARSCoV2^FL^-D614G, containing the prevalent A23403>G mutation, was generated by overlapping PCR, using plasmid pUC57-F5 (**Table 1**) as a template. First, a 655 bp PCR product was amplified with oligonucleotides WH-22763-VS (5’- GTAATTAGAGGTGATGAAG-3’) and D614G-RS (5’-CTGTGCAGTTAACA**C**CCTGATAAAGAACAGC-3’, A23403>G mutation in bold type), and a 1,788 bp PCR product was amplified using oligonucleotides D614G-VS (5’-GCTGTTCTTTATCAGG**G**TGTTAACTGCACAG-3’, A23403>G mutation in bold type) and WH-25174-RS (5’- TCCAAGTTCTTGGAGATCG-3’). Overlapping PCR product was then amplified using intermediate PCR products as templates and oligonucleotides WH-22763-VS and WH-25174-RS. The final 2,412 bp PCR product was digested with *Bst*EII and *Eco*RV and cloned into the same sites of pUC57-F5 (**Table 1**) to generate pUC57-F5-D614G. Finally, pUC57-F5-D614G was digested with *San*DI and *Bam*HI and cloned into the same sites of pBAC-SARSCoV2^FL^, to generate the pBAC-SARSCoV2^FL^-D614G. The genetic integrity of the cloned DNAs was verified throughout the subcloning and assembly process by extensive restriction analysis and sequencing.

### 2.6 Engineering of SARS-CoV-2 reporter replicon

To engineer a SARS-CoV-2-derived RNA replicon, the full-length infectious cDNA clone pBAC-SARSCoV2^FL^, as described above, was used as a backbone. A DNA fragment of 4,028 bp was chemically synthesized and purchased from GeneArt (Thermo Fisher Scientific, Germany). This fragment contained SARS-CoV-2 genome nt 20,085 to 21,556, fused to M gene transcription regulating sequence (TRS-M, nt 26,431 to 26,523) preceding mNeonGreen reporter gene (Shaner et al., 2013), and nt 28,177 to 29,870, including N gene and its TRS sequence. The synthetic DNA fragment was digested with *San*DI and *Rsr*II, and cloned into the same restriction sites of pBAC-SARSCoV2^FL^, leading to plasmid pBAC-SARSCoV2-REP-mNG.

To generate a non-replicative SARS-CoV-2 replicon as a control, plasmid pUC57-F2 (**Table 1**) was digested with *Nhe*I. The insert, containing nt 1,545 to 4,201, was cloned in pUC57-F2 in the reverse orientation, generating plasmid (pUC57-F2-NR). Finally, pUC57-F2-NR was digested with *Bsi*WI and *Pme*I, and cloned into the same restriction sites of pBAC-SARSCoV2-REP-mNG, leading to plasmid pBAC-SARSCoV2-REP-mNG-NR. All constructs were verified by extensive restriction analysis and sequencing.

### 2.7 Transfection of cells and recovery of rSARS-CoV-2 viruses

VeroE6-TMPRSS2 cells were grown to 95% confluence in F12.5 flasks. Cells were transfected with 4 μg of SARS-CoV-2 infectious cDNAs and 12 μl of Lipofectamine™ 2000 transfection reagent (Thermo Fisher Scientific) according to the manufacturer’s instructions and incubated at 37°C. At 72 hours post-transfection (hpt), the supernatant was collected (P0, passage 0) and stored at -80°C. Viruses were cloned from P0 by three rounds of plaque purification following standard procedures. Two selected viral clones were amplified to obtain viral stocks.

### 2.8 Analysis of virus stability

Cells were infected with either SARS-CoV-2-MAD6 virus or recombinant rSARS-CoV-2 viruses at an moi of 0.1. Up to nine blind passages were performed each 48 hpi. Supernatants were collected for titration and cells for RNA extraction. The loss of furin cleavage site was suggested by the appearance of a large plaque phenotype during plaque titration assay. In addition, total RNA from infected cells at different passages was isolated using RNeasy Mini Kit (Qiagen) according to the manufacturer’s instructions. Total cDNA was synthesized with random hexamers from 100 ng of total RNA as a template using High-Capacity cDNA reverse transcription kit (Applied Biosystems), following the manufacturer’s recommendations. A set of overlapping PCRs covering the whole SARS-CoV-2 genome was performed using oligonucleotides in **Supplementary Table 1**. PCR products were sequenced and sequences were assembled using SeqMan Ultra software from DNA Lasergene 17.3 package (Madison, WI).

### 2.9 Virus infection of mice or hamsters

Transgenic K18-hACE2 mice (Zheng et al., 2021) were maintained at the CNB-CSIC animal core facility. Six-week-old Syrian golden hamsters were provided by Envigo. All work with infected animals was performed in a BSL3+ laboratory at the Center for Animal Health Research (CISA, CSIC-INIA). Animals were anesthetized with 5% inhaled isoflurane for induction and 1.5-2% for maintenance, and intranasally inoculated with different doses of SARS-CoV-2 in DMEM. Weight loss and mortality were evaluated daily. Animals approaching 25% of weight loss were euthanized by 5% overdose of inhaled isoflurane until respiratory arrest for 90 seconds (Douglas et al., 2018). Three animals per group were euthanized as indicated above and necropsied at days 3 and 6 after inoculation, depending on the experiment. Lungs were collected and examined for macroscopic lesions. Lungs were then divided and were either frozen for subsequent virus titration, placed in RNAlater stabilization reagent (Life Technologies) for RNA extraction, or fixed in ready-to-use zinc formalin fixative (Z2902, Sigma-Aldrich) for histopathology. To determine SARS-CoV-2 titers, lungs were homogenized in phosphate-buffered saline (PBS) containing 100 IU/ml penicillin, 0.1mg/ml streptomycin, 50 µg/ml gentamicin, and 0.5 µg/ml amphotericin B (Fungizone), using a BeadBug 6 microtube homogenizer (Merck) and Lysing matrix S tubes (MPbio).

### 2.10 Analysis of RNA by quantitative RT-PCR

Total intracellular RNA was purified from cells or tissue samples with RNeasy Mini kit (Qiagen) according to the manufacturer’s instructions. Total cDNA was synthesized with random hexamers from 100 ng of total RNA as a template using High-Capacity cDNA reverse transcription kit (Applied Biosystems), following the manufacturer’s recommendations. SARS-CoV-2 viral RNA synthesis was analyzed using two custom TaqMan assays specific for gRNA (forward primer 5’- GTGARATGGTCATGTGTGGCGG-3’, reverse primer 5’- CARATGTTAAASACACTATTAGCATA-3’, and MGB probe 5’- CAGGTGGAACCTCATCAGGAGATGC-3’) and sgmRNA-N (forward primer 5’- CCAACCAACTTTCGATCTCTTGT-3’, reverse primer 5’- GGGTGCATTTCGCTGATTTT-3’, and MGB probe 5’- TTCTCTAAACGAACAAACTA-3’). Cellular gene expression was analyzed using specific TaqMan gene expression assays (**Table 2**) and hydroxymethylbilane synthase (HMBS) as a reference housekeeping gene. Data were acquired with a QuantStudio 5 real-time PCR system (Applied Biosystems) and analyzed with QuantStudio Desing & Analysis software v1.5.1. Relative quantifications were performed using the 2^-ΔΔCt^ method (Livak and Schmittgen, 2001). All experiments and data analysis were MIQE compliant (Bustin et al., 2009).

**Table 2:** TaqMan assays for cytokines.

GENE NAME ^(a)^	ASSAY ID ^(b)^	DESCRIPTION
CCL2	Hs00234140_m1	Monocyte chemotactic protein (MCP)-1
CCL4	Hs99999148_m1	Macrophage inflammatory protein (MIP)-1β
CXCL10	Hs00171042_m1	Interferon gamma induced protein 10 (IP-10)
HMBS	Hs00609297_m1	Hydroxymethylbilane synthase
IFNB1	Hs02621180_s1	Interferon beta (IFN-β)
IFNL3	Hs04193048_gH	Interferon lambda (IFN-λ) 3
IL-6	Hs00985641_m1	Interleukin 6
ISG15	Hs01921425_s1	Ubiquitin-like protein ISG15
MX1	Hs00895608_m1	Interferon-induced GTP-binding protein Mx1
TNF	Hs99999043_m1	Tumor necrosis factor alpha (TNFα)

^(a)^ In agreement with the Human Genome Organization (HUGO) Gene Nomenclature Committee (HGNC, https://www.genenames.org).

^(b)^ Gene expression assays were from Thermo Fisher Scientific.

Human-specific assays used to quantify cellular mRNAs by qPCR.

### 2.11 Immunofluorescence analysis

Cells grown on 13 mm glass coverslips were fixed by incubation with 4% paraformaldehyde in PBS for 40 min at room temperature. Cells were permeabilized with ice-cold methanol for 10 min and blocked with 10% FBS in PBS for 1h at room temperature. Primary antibodies were diluted in PBS with 5% FBS as follows: rabbit anti-ACE2 [EPR4436] (1:300, Abcam); rabbit anti-TMPRSS2 [EPR3862] (1:300, Abcam). Cells were then washed five times for 5 min each time with PBS and incubated for 30 min at room temperature with species-specific secondary antibodies conjugated to Alexa Fluor 488 or Alexa Fluor 594, diluted 1:500 in PBS with 5% FBS. Cell nuclei were stained with DAPI (1:200, Sigma). Finally, coverslips were mounted in Prolong Gold antifade reagent (Invitrogen). Confocal microscopy analysis was performed using a Leica SP5 laser scanning microscope, and images were collected and processed with LAS AF software (Leica, Wetzlar, Germany).

### 2.12 SARS-CoV-2 replicon activity analysis

Analysis of viral RNA synthesis by SARS-CoV-2 replicons was performed as previously described (Becares et al., 2016). Briefly, RNA was isolated from transfected cells at 48 hpt using RNeasy kit (Qiagen). DNA was removed from samples with DNaseI and cleaned RNA was subjected to RT-qPCR for the detection of viral RNAs as described above.

Reporter gene expression was detected by fluorescence microscopy using a Leica DMI6000B fluorescence microscope and OrcaR2 digital camera for image capture. Fiji software (Schindelin et al., 2012) was used for minimal adjustment to enhance the contrast for bright-field images and to merge them with the fluorescence images.

### 2.13 Statistical analysis

Two-tailed, unpaired Student’s *t* tests were used to analyze the difference in mean values between groups. All results were expressed as mean ± the standard deviation (SD) or standard error of the mean (SEM). *P* values <0.05 were considered significant.

## 3 Results

### 3.1 Engineering of SARS-CoV-2 infectious cDNA

**Figure 1:**
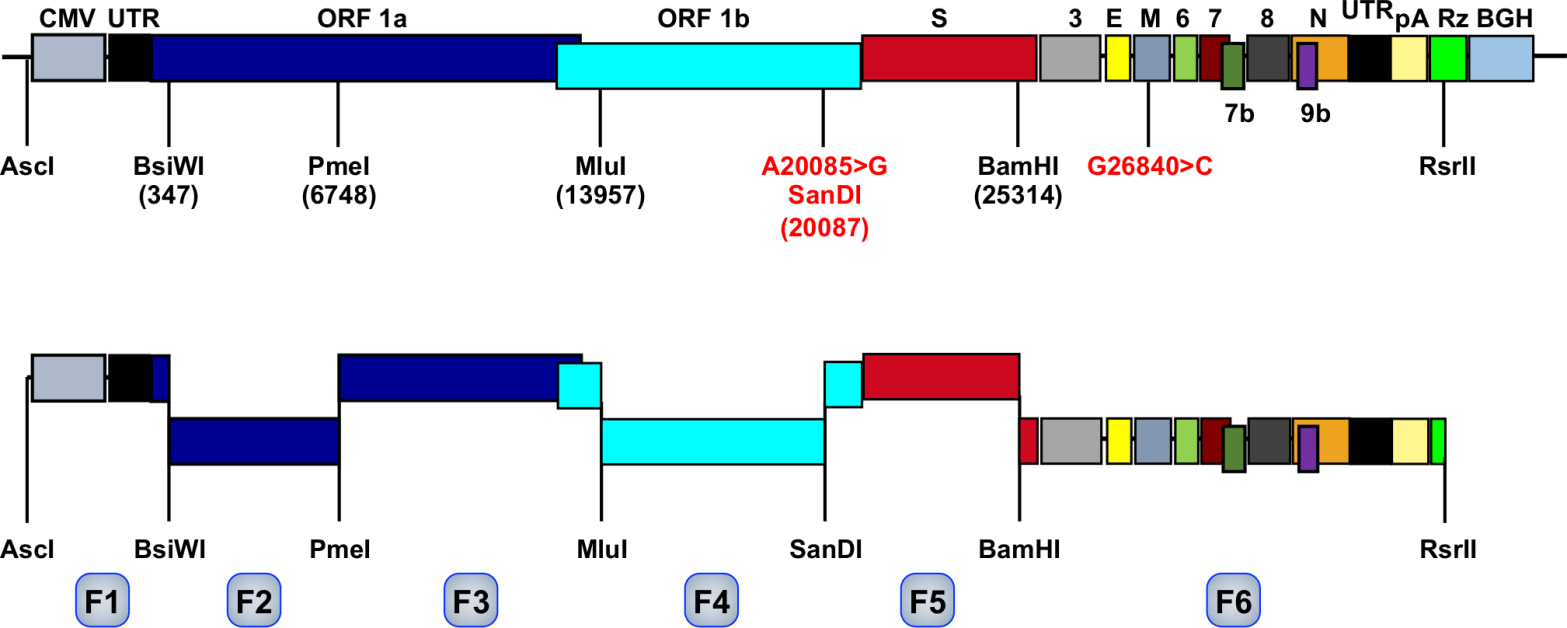
Assembly of SARS-CoV-2 infectious cDNA clone. The upper bar represents SARS-CoV-2 genome organization. Letters above the bar indicate the viral genes, flanked by cytomegalovirus promoter (CMV), and hepatitis delta virus ribozyme (Rz) and bovine growth hormone termination sequence (BGH). UTR, untranslated region. pA, poly (A) tail. Relevant restriction sites are indicated (position in the viral genome indicated by numbers in parenthesis) and the genetic markers introduced at position 20085 A to G to insert the SanDI restriction site and at position 26840 (G to C) are indicated in red. The bar below represents the six fragments (F1 to F6, light blue) designed to assemble SARS-CoV-2 infectious cDNA flanked by the selected restriction sites.

A SARS-CoV-2 infectious cDNA clone (pBAC-SARS-CoV-2^FL^) was engineered by sequential assembly of synthetic DNA fragments, covering the entire viral genome, using selected unique restriction sites (**Figure 1**). The cDNA clone sequence was identical to the reported Wuhan-Hu-1 isolate sequence (GenBank accession MN908947), with the exception of two silent point mutations: A20085>G, creating a unique *San*DI restriction site, and G26840>C, eliminating *Mlu*I and *Bsi*WI restriction sites. These point mutations were also used as genetic markers to distinguish between the virus recovered from the cDNA clone and a wild-type virus. By February 11^th^ 2023, there were more than five million SARS-CoV-2 genome sequences in the databases, but according to NextStrain analysis (Hadfield et al., 2018), the two engineered silent mutations were not present in the sequenced genomes, highlighting its usefulness as genetic markers.

**Figure 2:**
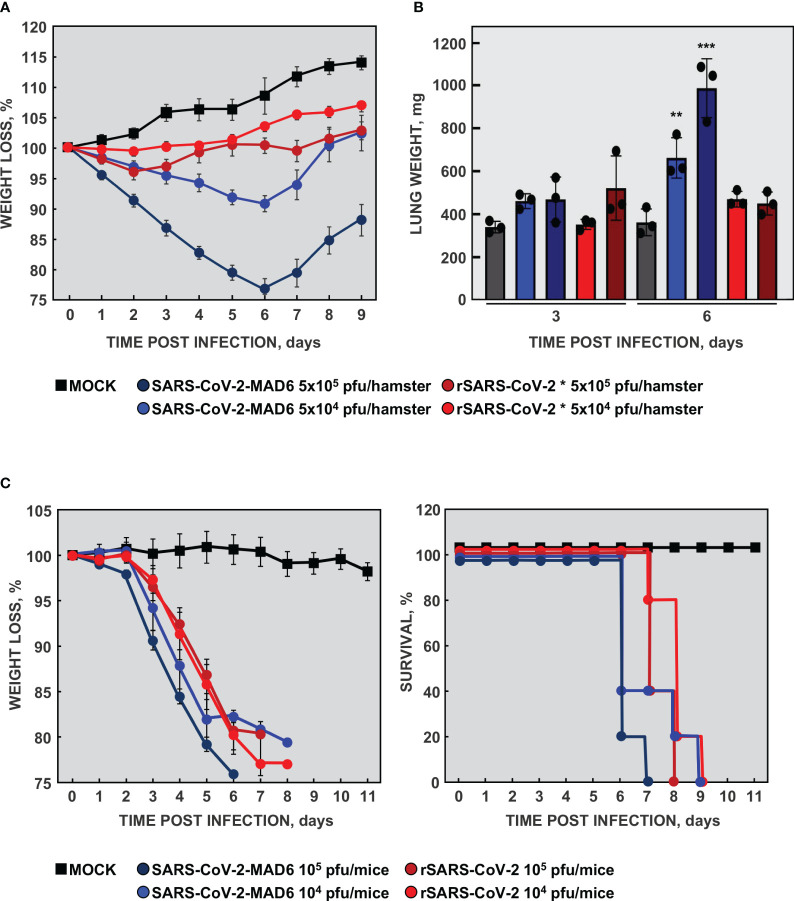
Virulence of recombinant SARS-CoV-2. **(A)** Virulence of the cell-culture adapted virus in the hamster model. Six-week-old Syrian golden hamsters were infected with two different doses of isolated SARS-CoV-2 MAD6 or recombinant cell-culture adapted rSARS-CoV-2 * viruses. Weight loss was monitored for nine days. The values represent means from four hamsters per group. Error bars indicate the SEM. **(B)** Three hamsters per group were sacrificed at 3 and 6 dpi and lungs were collected. Lung weight was measured as an indication of lung damage caused by edema and cell infiltration. The values in the column represent the mean for each group and the dots represent the individual animals. Error bars indicate the SEM. Compared with mock infected mice, p value, **, <0.01; ***, < 0.001. **(C)** Virulence of rSARS-CoV-2 virus in mice. 16-week-old K18-hACE2 mice were infected with two different doses of isolated SARS-CoV-2 MAD6 or recombinant rSARS-CoV-2 viruses. The weight loss (left panel) and survival (right panel) of the mice were monitored for 11 days. The values represent means from five mice per group. Error bars indicate the SEM.

The recombinant rSARS-CoV-2 virus was efficiently recovered from the infectious cDNA with a titer of 6×10^6^ pfu/ml. However, a mutated virus with a large plaque phenotype that emerged in passage 1 (rSARS-CoV-2 *), grew to up to 15-fold higher titers and outcompeted the wild-type virus within three passages on VeroE6 cells. Sequencing results revealed that the mutant virus contained a 15-nt deletion from nt 23583 to 23597 in the S gene. This deletion eliminated five amino acids (QTQTN) upstream to the furin cleavage site. Similar deletions, leading to an attenuated virus, were previously described (Lau et al., 2020). Using the best available animal model at that time, the hamster model, we observed that, in fact, the mutant virus adapted to VeroE6 cells was attenuated, causing no weight loss (**Figure 2A**) or macroscopic damage measured as lung weight increase (**Figure 2B**), in contrast with the patient-isolated SARS-CoV-2-MAD6 virus. These data were in agreement with published observations indicating that mutations close to or eliminating the furin cleavage site attenuate SARS-CoV-2 (Johnson et al., 2021; Vu et al., 2022).

To avoid virus instability, rSARS-CoV-2 virus was recovered using human Calu3 2B4 cells (Yoshikawa et al., 2010) and monkey VeroE6 cells overexpressing human TMPRSS2 protease (VeroE6-TMPRSS2). Recovered virus reached titers of 2×10^6^ pfu/ml and 1×10^7^ pfu/ml in Calu3 2B4 and VeroE6-TMPRSS2 cells, respectively. The rSARS-CoV-2 virus genome was fully sequenced and no genetic changes were identified. Moreover, in both cell lines the virus was fully stable for at least nine passages, maintaining an intact furin cleavage site.

The transgenic K18-hACE2 mice model was then available, being a better lethal model for SARS-CoV-2 infection than hamster (Zheng et al., 2021). To analyze the virulence of the engineered rSARS-CoV-2 virus, 16-week-old K18-hACE2 mice were infected with two different doses of isolate SARS-CoV-2-MAD6 or of recombinant rSARS-CoV-2 virus recovered from Calu3 2B4 cells. The mice infected with SARS-CoV-2 MAD6 and rSARS-CoV-2 viruses significantly lost weight compared with mock-infected mice (**Figure 2C**). In addition, all the mice infected with both isolated SARS-CoV-2-MAD6 and recombinant rSARS-CoV-2 viruses died between days 6 and 9 post-infection, depending on the inoculated dose (**Figure 2C**). These data indicated that recombinant rSARS-CoV-2 virus, recovered from the infectious cDNA, was virulent in K18-hACE2 mice.

### 3.2 Improvement of cell culture system for SARS-CoV-2

**Figure 3:**
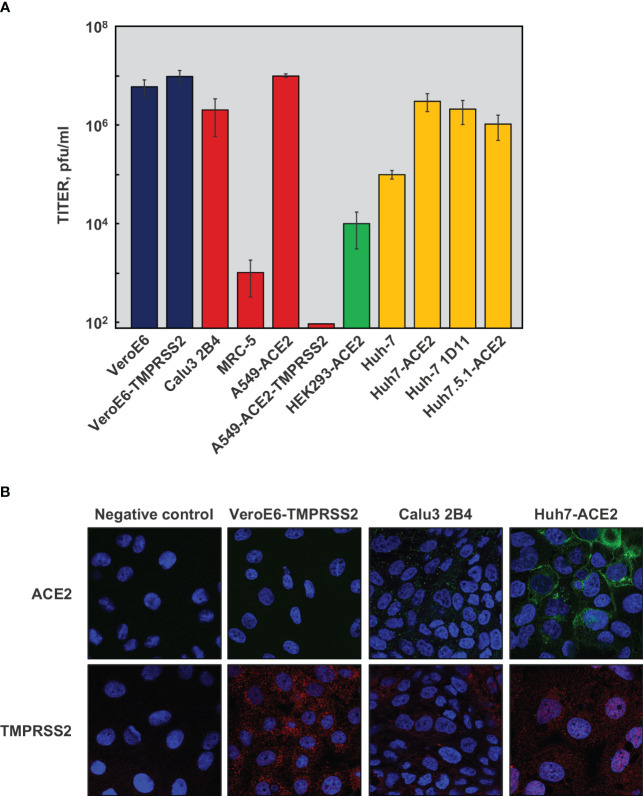
Culture of SARS-CoV-2 in different cell lines. **(A)** Eleven cell lines from different origin were infected with SARS-CoV-2 at a MOI of 0.1. Monkey cells (blue), and human cells derived from lung (red), kidney (green) or liver (yellow) were tested. Culture supernatant samples were harvested at 48 hpi and titrated on VeroE6 cells by plaque assay. Viral titers indicate the mean from three independent infections; error bars represent SDs. **(B)** Immunofluorescence detection of human ACE2 (green, upper panels) and TMPRSS2 (red, bottom panels). Merge layer including cell nuclei, stained with DAPI (blue), are shown. Negative control was performed without primary antibodies, to set up the background with only secondary antibodies.

As indicated above, genomic instability in SARS-CoV-2 was observed when the virus was passaged in VeroE6 cells, with deletions appearing in the S protein region close to the furin cleavage site. Calu3 2B4 cells are difficult to culture in monolayer and other authors have extensively used alternative cells lines for SARS-CoV-2 infection, although virus stability was not systematically tested in these cells. To improve the cell culture system, eleven human and non-human cell lines were tested. Seven of them were susceptible to SARS-CoV-2 infection (**Figure 3A**). Among these susceptible cell lines, SARS-CoV-2 growth was more robust in VeroE6-TMPRSS2, Huh-7-ACE2 and Calu3 2B4 cells with titers of 1×10^7^ pfu/ml, 3×10^6^ pfu/ml and 2×10^6^ pfu/ml, respectively. Moreover, virus sequencing indicated that the furin cleavage site was maintained at least for six passages (**Table 3**). Significant virus production was also obtained in A549-ACE2, VeroE6 and Huh-7.5.1-ACE2 cells, with virus titers of 1×10^7^ pfu/ml, 6×10^6^ pfu/ml and 1×10^6^ pfu/ml, respectively. However, the furin cleavage site was not maintained during the passages in these cell lines (**Table 3**). Huh-7.5.1 cells are derived from Huh-7.5 cells, both containing more than 50 mutations compared with parental Huh-7 cells, all of the changes being adaptive mutations for better hepatitis C virus (HCV) growth (Appel et al., 2005; Zhong et al., 2005; Kawamoto et al., 2020). This may explain the differences in virus stability observed between the two cell lines. Surprisingly, virus replicated efficiently in A549-ACE2-TMPRSS2 cells as determined by RT-qPCR (**Supplementary Figure 1**), but no infectious virus was detected by plaque assays (**Figure 3A**).

**Table 3:** SARS-CoV-2 stability of S protein furin cleavage domain in different cell lines during virus passage.

CELL LINE	TYPE	TITER, pfu/ml	STABILITY
VeroE6	Monkey kidney	(6.0±1.1) x10^6^	No
VeroE6-TMPRSS2	Monkey kidney	(1.0±0.7) x10^7^	Yes
Calu3 2B4	Human lung adenocarcinome	(2.3±1.5) x10^6^	Yes
A549-ACE2	Human lung adenocarcinome	(1.0±0.5) x10^7^	No
Huh7-ACE2	Human hepatocarcinome	(3.1±1.4) x10^6^	Yes
Huh-7 1D11	Human hepatocarcinome	(1.0±2.0) x10^6^	Yes
Huh7.7.1-ACE2	Human hepatocarcinome	(0.9±2.2) x10^6^	No

It has been reported that SARS-CoV-2 infection and genetic stability required the expression of both human ACE2 receptor and TMPRSS2 protease (Hoffmann et al., 2020b; Peacock et al., 2021). The levels of these proteins were analyzed by immunofluorescence in VeroE6-TMPRSS2, Calu3 2B4 and Huh-7-ACE2 cells. As expected, human ACE2 was not detected in VeroE6-TMPRSS2 cells, while it was expressed in human cells (**Figure 3B**, upper panels). TMPRSS2 protease was expressed in all cell lines, although nuclear and cytoplasmic location in Huh-7-ACE2 cells differed from the exclusive cytoplasmic location observed in the other two cell lines (**Figure 3B**, lower panels). Similar cell-type dependent TMPRSS2 subcellular pattern was previously reported for other cell types and may have influence in cell susceptibility to the infection (Bertram et al., 2013; Thul et al., 2017).

### 3.3 Effect of D614G mutation in SARS-CoV-2 virus growth

**Figure 4:**
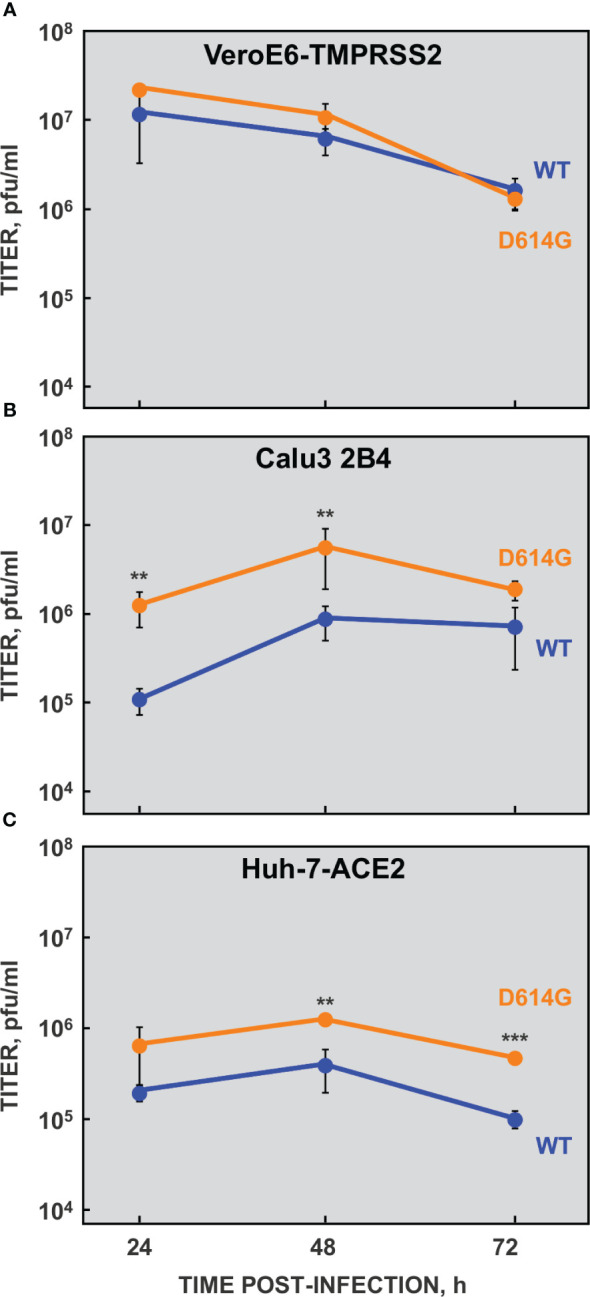
Growth kinetics of rSARS-CoV-2-D614G mutant virus. VeroE6-TMPRSS2 **(A)**, Calu3 2B4 **(B)** and Huh-7-ACE2 **(C)** cells were infected at a MOI of 0.1 with rSARS-CoV-2-D614G (D614G, orange) or parental (WT, blue) viruses. Cell culture supernatants were harvested at 24, 48 and 72 hpi and virus titers were determined by plaque assay on VeroE6 cells. The data indicate the mean from three independent infections; error bars represent SDs. P value, **, <0.01; ***, < 0.001.

**Figure 5:**
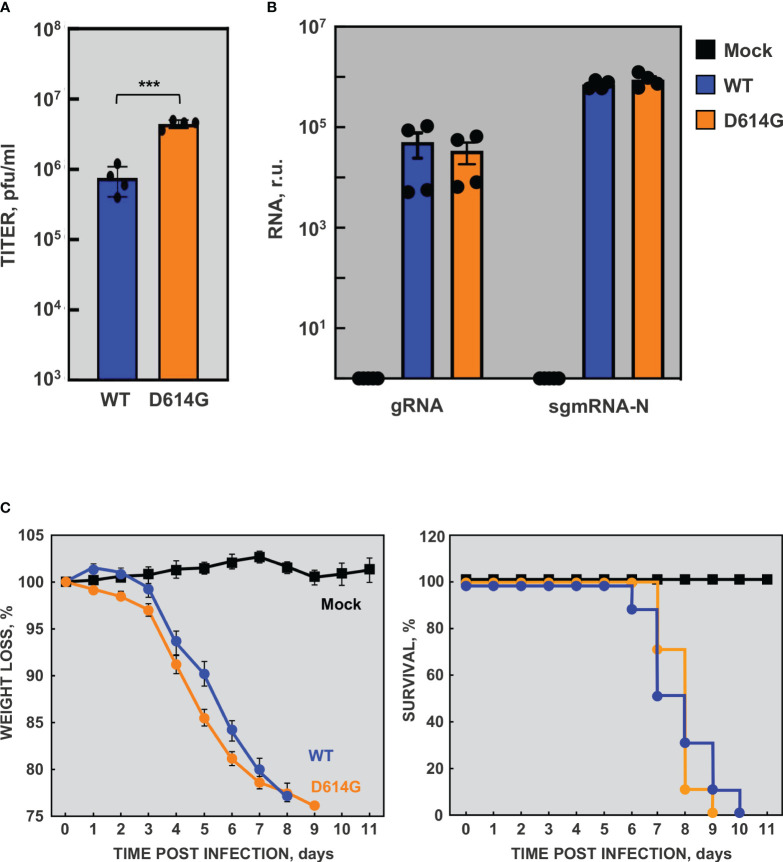
Viral RNA synthesis and virulence by rSARS-CoV-2-D614G mutant virus. Calu 3 2B4 cells were mock infected (Mock) or infected with rSARS-CoV-2 (WT, blue) and rSARS-CoV-2-D614G (D614G, orange) viruses at a MOI of 1. At 16 hpi, supernatants were collected and virus titers were determined **(A)**. In addition, total RNA was extracted and the levels of gRNA and sgmRNA-N were determined by RT-qPCR **(B)**. RNA levels were normalized by HMBS mRNA levels. In addition, sgmRNA-N levels were made relative to gRNA levels. r.u., relative units. The data indicate the mean from four independent infections. Error bars indicate the SEM. P value, ***, < 0.001. **(C)** Virulence of rSARS-CoV-2 virus. 24-week-old K18-hACE2 mice were infected with 10^4^ pfu/mouse of rSARS-CoV-2 (WT, blue) or rSARS-CoV-2-D614G (D614G, orange) viruses. The weight loss (left panel) and survival (right panel) of the mice were monitored for 11 days. The values represent means from ten mice per group. Error bars indicate the SEM.

A slight delay in weight loss and lethality was observed in susceptible transgenic mice infected with the rSARS-CoV-2-WH1 virus compared with those infected with isolate SARS-CoV-2-MAD6 virus (**Figure 2C**). The genomes of these two viruses were identical except for the two genetic markers and three-point mutations: the silent mutation C3037>T and two missense mutations C14408>T (affecting nsp12) and A23403>G (leading to D614G in S protein). Interestingly, the S protein D614G mutation became prevalent in SARS-CoV-2 isolates early during the global pandemic and it has been maintained in all SARS-CoV-2 variants since then (Antoneli et al., 2021; Chakraborty et al., 2021). To further characterize the cell culture system for SARS-CoV-2, based on our previous observation and given its rapid rise in human isolates and enhanced transmission, we engineered rSARS-CoV-2-D614G mutant virus as a more representative virus compared with the parental one. The genome of the rSARS-CoV-2-D614G mutant virus was identical to that of the parental rSARS-CoV-2 virus except for the point mutation A23403>G. Viral growth kinetics of rSARS-CoV-2-D614G mutant virus was determined in VeroE6-TMPRSS2 (**Figure 4A**), Calu3 2B4 (**Figure 4B**), and Huh-7-ACE2 (**Figure 4C**). No significant differences were observed in VeroE6-TMPRSS2 cells between the mutant and parental viruses. In contrast, the viral titers of D614G variant were significantly higher (up to 10-fold) than those of the parental virus in human cells, both Calu3 2B4 and Huh-7-ACE2 cells. These data were in agreement with previous observations (Hou et al., 2020; Yurkovetskiy et al., 2020; Zhang et al., 2020; Plante et al., 2021; Zhou et al., 2021). The increased viral titer (**Figure 5A**) was not due to differences in viral RNA synthesis, as the accumulation of gRNA and sgmRNA-N was similar in rSARS-CoV-2-D614G and parental virus infected Calu3 2B4 cells (**Figure 5B**). The rSARS-CoV-2-D614G mutant virus was virulent in K18-hACE2 mice with a slightly accelerated weight loss compared with the rSARS-CoV-2 virus, as expected (**Figure 5C**). Both viruses had similar virulence measured as mice survival, in line with published data (Korber et al., 2020; Stauft et al., 2021).

### 3.4 Cytokine expression in infected human cell lines

**Figure 6:**
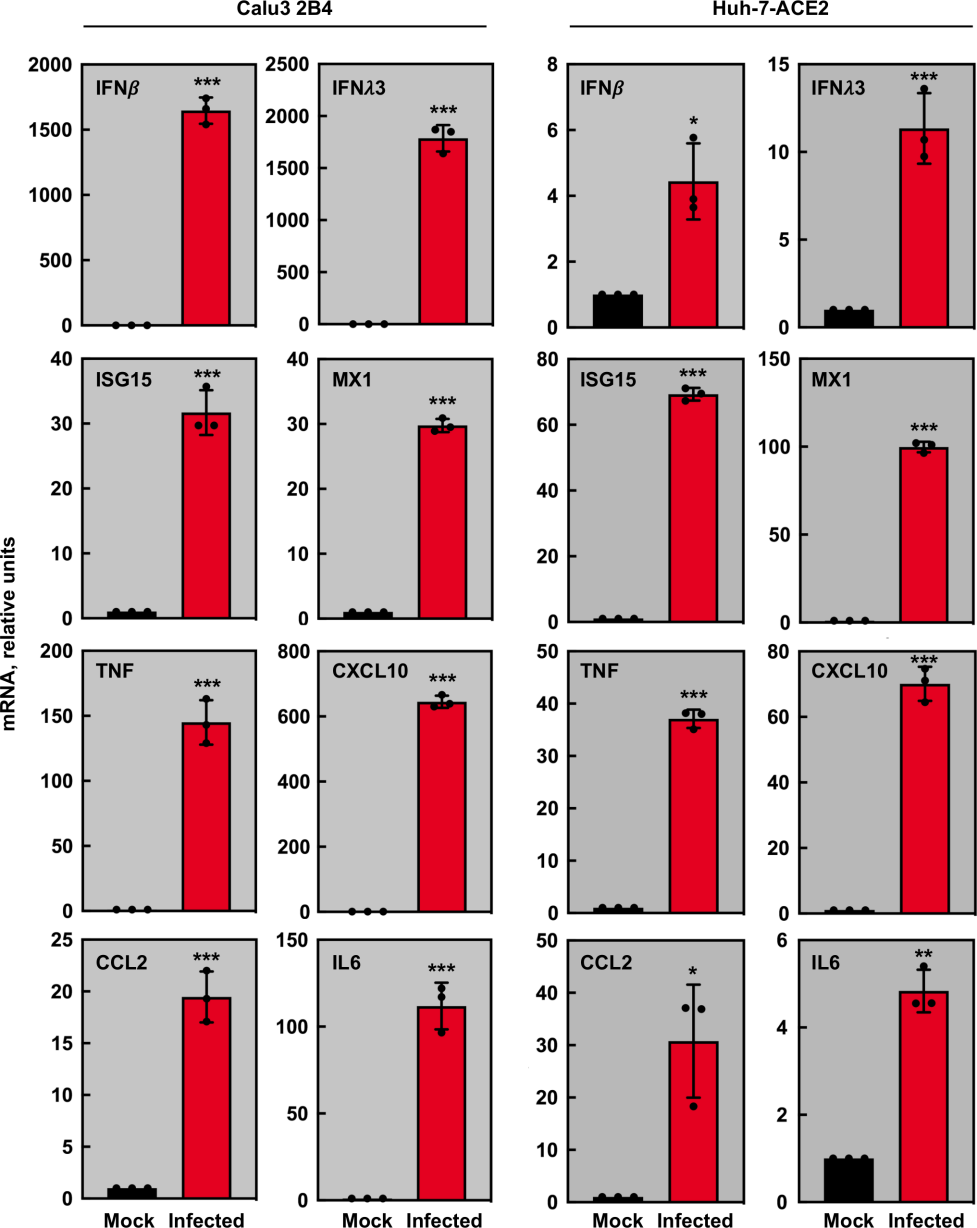
Cytokine mRNA accumulation in rSARS-CoV-2-D614G infected cells. Calu3 2B4 or Huh-7-ACE2 cells were mock-infected (black) or infected at a MOI of 1 with mutant rSARS-CoV-2-D614G (red) virus. Quantification of mRNAs encoding IFN-β, IFN-Λ, ISG15, MX1, TNF, IL6, CCL2, and CXCL-10 was performed by RT-qPCR using specific TaqMan assays. The HMBS mRNA was used as a reference gene; relative mRNA levels were based on the comparison with mock-infected cells. The data represent the mean from three different infections. Error bars indicate SDs. Compared with mock infected cells, p value, *, <0.05; **, < 0.01, ***, < 0.001.

Based on previous data from *in vivo* models mimicking SARS-CoV-2 infection in humans (Zheng et al., 2021; Wong et al., 2022), a panel of cytokines was selected to analyze their mRNA accumulation in cells infected with rSARS-CoV-2-D614G virus. From the previously selected cell lines, only the cytokine expression in human Calu3 2B4 and Huh-7-ACE2 was evaluated, since VeroE6-TMPRSS2 cells are defective in the innate immune response. The levels of genes related with IFN response, such as IFN-β, IFN-λ, or IFN stimulated genes (ISGs) such as ISG15 and MX1, and the levels of pro-inflammatory cytokines, such as TNF, CCL2, CXCL10 or IL-6 were evaluated by RT-qPCR (**Figure 5**). mRNAs for these genes were significantly increased in both infected Calu3 2B4 and Huh-7-ACE2 cells as compared with mock-infected cells. Nevertheless, the cytokine accumulation pattern observed in infected Calu3 2B4 mimicked the one observed *in vivo*, both in mouse models (Zheng et al., 2021; Wong et al., 2022) or in patients (Mudd et al., 2020; Galani et al., 2021; Tang et al., 2021), while cytokine increases were lower in infected Huh-7-ACE2 cells (**Figure 6**).

Altogether, the data indicated that both VeroE6-TMPRSS2 and Calu3 2B4 cells were an adequate cell culture system to amplify SARS-CoV-2 virus, maintaining viral genetic stability and virulence. In addition, innate immune responses in Calu3 2B4 cell line resembled those observed in animal models and humans, making this cells line optimal for virus-host interaction studies.

### 3.5 Engineering of a SARS-CoV-2 reporter replicon

**Figure 7:**
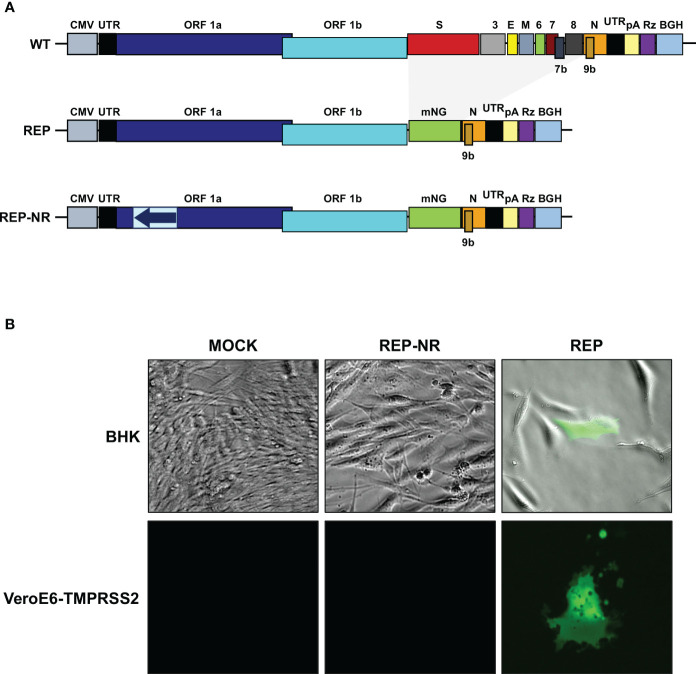
Engineering of SARS-CoV-2 reporter replicon. **(A)** Scheme of the SARS-CoV-2 cDNA (upper panel) and replicons (middle and bottom panels) clones in BACs. The letters above the boxes indicate the viral genes or the reporter gene mNeonGreen (mNG). The non-replicative control replicon (REP-NR) contains a fragment comprising nt 1,545 to 4,201 of the viral genome in the reverse orientation (blue arrow in light blue box). UTR, untranslated region; CMV, cytomegalovirus promoter; pA, polyA sequence; Rz, hepatitis delta virus ribozyme; BGH, bovine growth hormone polyadenylation and termination signals. **(B)** BHK-21 (upper panels) and VeroE6-TMPRSS2 cells (lower panels) were mock transfected or transfected with non-replicative control replicon (REP-NR) or SARS-CoV-2 replicon (REP). Fluorescence was analyzed at 48 hpt. Merge of representative bright-field and fluorescence images are shown in the upper panel to illustrate the absence of fluorescence in neighboring cells, while representative fluorescence images are shown in the lower panel to illustrate cell fusion.

The availability of a SARS-CoV-2 replicon provides an important tool for the study of fundamental viral processes and the screening of antiviral drugs in the absence of infectious virus, not requiring BSL-3 conditions. Therefore, a SARS-CoV-2-derived replicon was constructed (pBAC-SARSCoV2-REP-mNG) derived from the full-length infectious cDNA clone as described above (**Figure 7**). This plasmid retained the untranslated 5’ and 3’ ends of the viral genome, the replicase genes, and the N gene, which is required for efficient CoV RNA synthesis (Almazan et al., 2004). A mNeonGreen (mNG) reporter gene was also introduced in the replicon plasmid, under the control of M gene TRS (Shaner et al., 2013). Since first amplification of replicon RNA comes from CMV promoter, a non-replicative RNA replicon was also engineered as negative control of viral RNA polymerase-dependent RNA synthesis (**Figure 7A**).

To evaluate the SARS-CoV-2 replicon functionality, the replicon was transfected into BHK (easily transfected cell line often used for CoV replicons analysis) or VeroE6-TMPRSS2 cells. Reporter protein expression was tested by fluorescence microscopy at 48 hpt. The mNG protein was expressed to high levels, compared with the non-transfected cells and the cells transfected with the non-replicating replicon (**Figure 7B**). Fluorescence was observed in isolated BHK cells, and in isolated foci of VeroE6-TMPRSS2 fused cells, demonstrating the inability of the replicon to propagate (**Figure 7B**).

**Figure 8:**
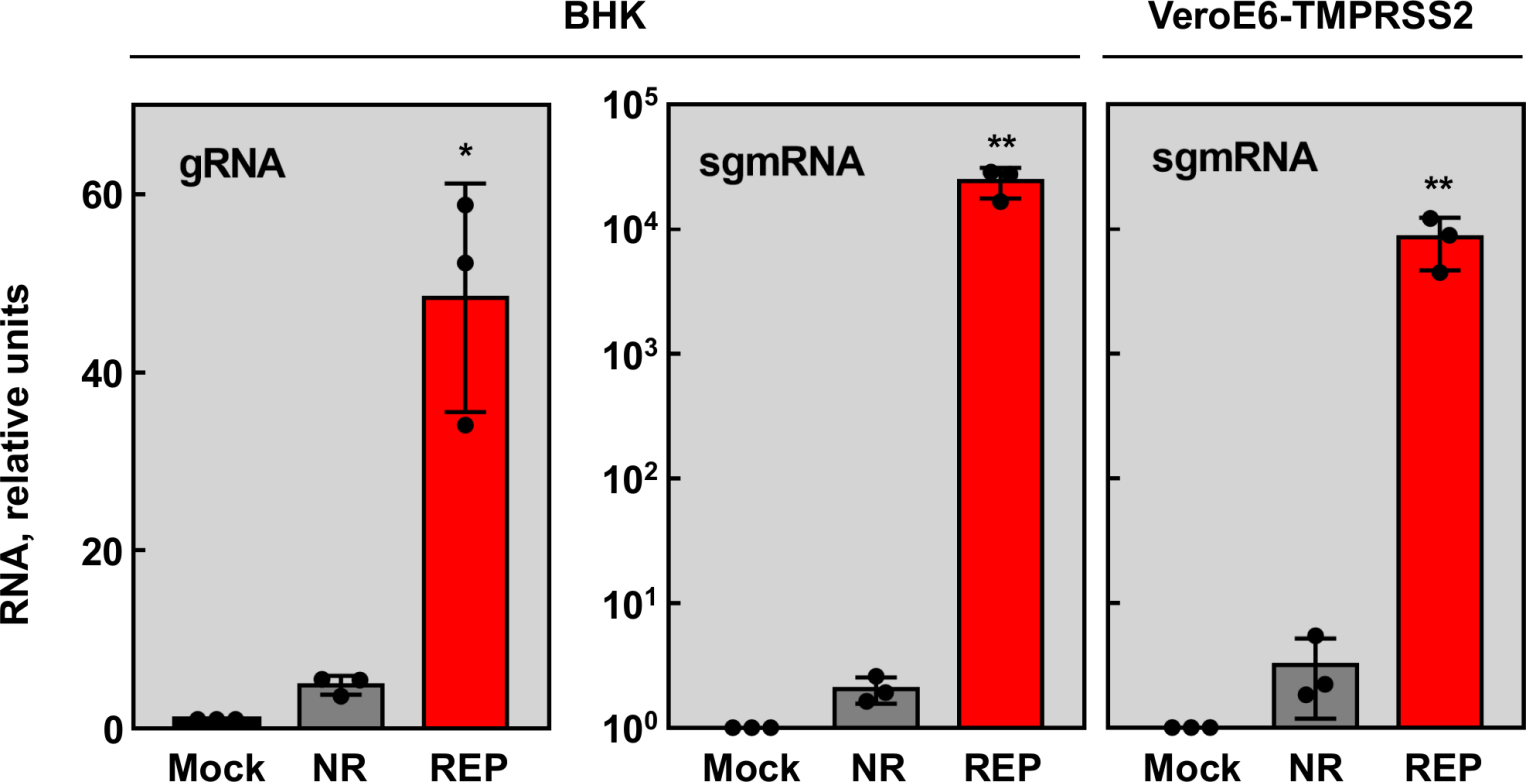
RNA synthesis by SARS-CoV-2 reporter replicon. BHK-21 and VeroE6-TMPRSS2 cells were mock transfected (Mock, black columns) or transfected with non-replicative control replicon (NR, gray columns) or SARS-CoV-2 replicon (REP, red columns). Total RNA was extracted at 48 hpt and replication (gRNA) and transcription (sgmRNA) levels were analyzed by RT-qPCR. The RNA levels were relative to those in the mock transfected cells. The values represent the mean of three independent transfections. Error bars indicate SDs. Compared with non-replicative control, p value, *, <0.05; **, < 0.01.

Viral RNA synthesis was analyzed by RT-qPCR. Both gRNA and sgmRNA-N accumulated significantly in cells transfected with the SARS-CoV-2 replicon, compared with the non-replicative replicon control (**Figure 8**). Altogether, the results indicated that SARS-CoV-2 replication-transcription complex was assembled, and viral RNAs and proteins were efficiently synthetized.

## 4 Discussion

In this work, we applied our reverse genetics approach based on the use of BACs to assemble a SARS-CoV-2 full-length cDNA (Almazan et al., 2000; Almazan et al., 2013; Almazan et al., 2015), leading to the recovery of a virulent rSARS-CoV-2 virus. Other systems to engineer CoV cDNA clones have also been developed, including SARS-CoV-2 (Thi Nhu Thao et al., 2020; Xie et al., 2020; Torii et al., 2021; McGrath et al., 2022; Melade et al., 2022). The advantages of our engineered BAC system include: (i) it permits the stable maintenance in bacteria of large DNA fragments (Shizuya et al., 1992; Adler et al., 2003; Ye et al., 2020); (ii) the manipulation of the cDNA clone is similar to that of a conventional plasmid; and (iii) it directly allows the recovery of infectious virus from the cDNA clone with no need for *in vitro* ligation and transcription steps, which usually have low efficiencies and yields. Our SARS-CoV-2 infectious cDNA clone has been made available, since early in the pandemic, to the scientific community worldwide, and it is being successfully used for further studies to improve SARS-CoV-2 animal models, to set up antiviral testing systems, or to analyze virulence factors (Malicoat et al., 2022; Wong et al., 2022; Alhammad et al., 2023; Honrubia et al., 2023).

During the rescue of a rSARS-CoV-2 virus from the infectious cDNA clone in VeroE6 cells, commonly used to grow SARS-CoV-2, deletions close to furin cleavage site were incorporated, and standard plaque size was changed by a large plaque phenotype, the evolved virus grew to higher titers and replaced the wild-type virus within one to four passages, and became attenuated *in vivo*. These results were in agreement with previous findings (Lau et al., 2020; Johnson et al., 2021; Vu et al., 2022). The SARS-CoV-2 furin cleavage site enhances serine protease-mediated entry, the dominant entry pathway in human airway cells in which membrane fusion occurs at the cell surface (Hoffmann et al., 2020b; Lamers et al., 2021; Mykytyn et al., 2021). Our data showed that virus propagation in human Calu3 2B4 cells, naturally expressing ACE2 and serine proteases, prevented furin cleavage site deletions leading to a virulent rSARS-CoV-2 virus. It has been suggested that VeroE6 cells lack the dominant entry pathway, forcing the virus to use the endosomal pathway, in which viral particles are internalized and membrane fusion occurs at the endosome, leading to furin cleavage loss (Peacock et al., 2021). It was proposed that the deletions in the furin cleavage site could be prevented in cells with an active serine protease-mediated entry pathway, such as those expressing TMPRSS2 serine protease (Bestle et al., 2020; Jackson et al., 2022). In agreement with this observation, we found that SARS-CoV-2 genetic stability was maintained for at least nine passages in VeroE6 cells stably expressing TMPRSS2.

Our results indicated that the higher viral titers were obtained in several human cell lines over-expressing ACE2, but furin cleavage site instability was observed in some cases. It was previously reported that SARS-CoV-2 efficiently infects cells that express ACE2 or co-express ACE2 and TMPRSS2 (Sungnak et al., 2020). In contrast with the published data, we found that A549 cells over-expressing ACE2 were efficiently infected and virus grew to high titers, but the virus acquired with passage deletions close to furin cleavage site. Over-expression of TMPRSS2 in these cells was not a solution for furin site instability, as SARS-CoV-2 infected A549 cells co-expressing ACE2 and TMPRSS2, but no virus production was observed. There are previous reports of SARS-CoV-2 infection of A549-ACE2-TMPRSS2 cells (Widera et al., 2021; Lenart et al., 2023; Vanderlinden et al., 2023). Interestingly, in most of these works, virus infection was monitored by viral RNA determination using RT-qPCR, obtaining similar levels of viral RNA as those observed by us (**Supplementary Figure 1**). It should be noted that active viral replication does not necessarily imply efficient morphogenesis and egress of infectious virus particles, although cytopathic effect can be observed due to cell death and release of cellular factors. In fact, in our laboratory similar observations were done for MERS-CoV, another pathogenic human CoV, which entered into A549 cells synthetizing viral RNAs with no release of infectious virus in the culture supernatant. These differences with previously published data may be due to different virus/cell systems and further studies are needed to clarify this issue.

An increased viral replication was observed for the rSARS-CoV-2-D614G virus, compared with the original rSARS-CoV-2-WH1, in agreement with previous studies demonstrating the enhanced virus replication of D614G variant, both *in vitro* and *in vivo* (Hou et al., 2020; Plante et al., 2021; Stauft et al., 2021), although the underlying molecular mechanism is not fully understood. Many studies used pseudotyped viral particles and proposed that there could be some differences in the proteolytic processing and incorporation of S D614G protein, compared with the original S protein (Yurkovetskiy et al., 2020; Zhang et al., 2020; Daniloski et al., 2021). However, two studies that used isogenic SARS-CoV-2 D614 and G614 variants found no difference in S protein cleavage and incorporation into viral particles (Hou et al., 2020; Plante et al., 2021). This discrepancy could most likely be due to differences in S protein trimer assembly and presentation in the pseudotyped virus systems. In fact, structural studies suggest that D614G mutation increases the stability of the S trimer, enhancing the infectivity of D614G variant viruses (Zhang et al., 2021).

Despite the higher viral titers of rSARS-CoV-2-D614G mutant virus in different human cell types, no significant differences were observed in the accumulation of viral gRNA and sgmRNA-N compared with the parental virus. This was in contrast with previous results describing a modest increase in gRNA levels for D614G mutant viruses (Plante et al., 2021). Technical differences in the assays used to quantify viral gRNA may explain this discrepancy. In our case specific TaqMan assays were used to specifically detect gRNA or sgmRNA-N, while in Plante et al. (2021), the RT-qPCR assay targets ORF8 therefore detecting gRNA and seven sgmRNAs together.

A slight increase in weight loss was observed in mice infected with rSARS-CoV-2-D614G mutant. No significant association between D614G mutation and disease severity, measured by hospitalization outcomes, was described (Korber et al., 2020). Moreover, no differences in pathogenicity of D614G and WT viruses were found in a hamster model (Stauft et al., 2021). Therefore, it could be suggested that the described slight differences may be associated with higher viral loads but not with direct modulation of pathogenesis mechanisms.

A SARS-CoV-2-derived replicon was also generated from the full-length cDNA clone, including a mNG reporter gene. The engineered SARS-CoV-2 replicon may be very useful for the identification of viral and cellular factors involved in CoV transcription and replication, for the safe screening of new antiviral drugs, and for the development of SARS-CoV-2 vaccines. Alternative strategies for the construction of SARS-CoV-2 replicons have also been reported, such as the use of a system based on several plasmids followed by *in vitro* DNA ligation of cDNA fragments (Xia et al., 2020; Kotaki et al., 2021), assembly in yeast (Ricardo-Lax et al., 2021), or based on a single BAC plasmid system (He et al., 2021; Fahnoe et al., 2022). The design of these replicons differed in the structural and accessory gene deletions and the reporter genes introduced. Our SARS-CoV-2 replicon, compared to most of these reported replicon systems, exhibits increased safety due to the large genome deletions, high stability, easy manipulation for large quantity production, and direct replicon activity evaluation after transfection with no need for *in vitro* ligation or transcription. In addition, since the non-replicative control replicon was also engineered, our system allows accurate evaluation of SARS-CoV-2 RNA synthesis eliminating the replicon RNA background produced by CMV promoter after cell transfection. In fact, our replicon system was successfully used for analysis of antiviral strategies (Hussein et al., 2023).

After transfection of VeroE6-TMPRSS2 cells, with the engineered replicon, we observed syncytia formation of significantly smaller size than the ones induced by SARS-CoV-2 infection (**Supplementary Figure 2**). This observation was unexpected, since the reporter replicon does not express S protein. These data are in line with previous observation indicating that syncytia formation by SARS-CoV-2 can be mediated by cell factors such as interferon-induced transmembrane proteins (IFITs) and TMPRSS2, independent of S protein (Buchrieser et al., 2020). Alternatively, a small fraction of N protein may be located in the cell membrane, as previously described in infected cells (Lopez-Munoz et al., 2022), facilitating cell fusion mediated by TMPRSS2.

The engineering of reverse genetics tools, such as the infectious cDNA and reporter replicon described in this work is essential to understand the functions of viral proteins and virus-host interactions modulating pathogenesis, and would help to screen and identify novel targets for antiviral strategies.

## Data availability statement

The original contributions presented in the study are included in the article/**Supplementary Material**. Further inquiries can be directed to the corresponding authors.

## Ethics statement

Experiments involving animals were performed in strict accordance with EU (2010/63/UE) and Spanish (RD 53/2013 and 32/2007) guidelines. All the protocols were approved by the on-site Ethical Committee (permit n°. PROEX 146.6/20). Infected mice were housed in a self-contained ventilated rack (Allentown, NJ).

## Author contributions

LW: Conceptualization, Investigation, Methodology, Validation, Writing – original draft, Writing – review & editing. MG: Conceptualization, Investigation, Methodology, Validation, Writing – original draft, Writing – review & editing. DM-S: Conceptualization, Investigation, Methodology, Validation, Writing – review & editing. JH: Investigation, Methodology, Validation, Writing – review & editing. JR-G: Investigation, Methodology, Validation, Writing – review & editing. RD: Resources, Writing – review & editing. IS: Funding acquisition, Writing – review & editing. LE: Conceptualization, Funding acquisition, Writing – original draft, Writing – review & editing. SZ: Conceptualization, Investigation, Methodology, Validation, Writing – original draft, Writing – review & editing.
