# Supplementary material for: Cell type dependent stability and virulence of a recombinant SARS-CoV-2, and engineering of a propagation deficient RNA replicon to analyze virus RNA synthesis
Source: Front Cell Infect Microbiol. 2023 Oct 24;13:1268227. doi: 10.3389/fcimb.2023.1268227 (PMC10628495; doi:10.3389/fcimb.2023.1268227)
Supplement: Supplementary file 1 [file DataSheet_1.pdf]

Supplementary Figure 1

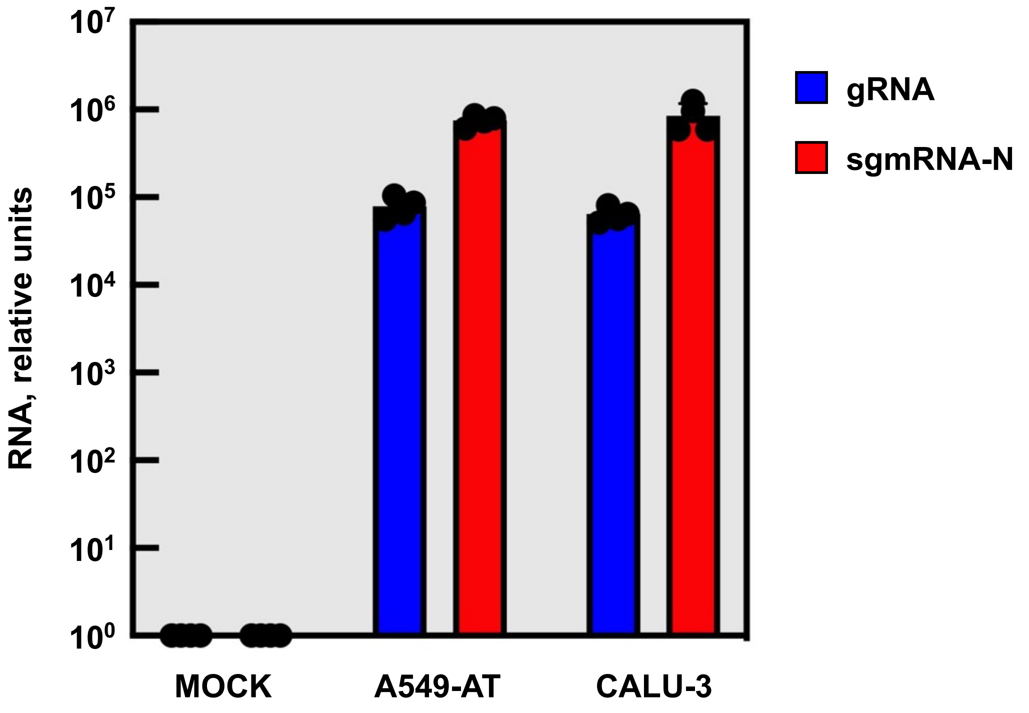

Supplementary Figure 1. Viral RNA accumulation in A549-ACE2-TMPRSS2 cells. A549-ACE2-TMPRSS2 cells (A549-AT) were either mock infected or infected with rSARS-CoV-2 virus at a moi of 0.1. Calu3 2B4 cells (CALU-3) were also infected as a control. At 24 hpi total RNA was extracted and the levels of gRNA (blue) and sgmRNA-N (red) were determined by RT-qPCR. The values represent means from four independent infections.

Supplementary Figure 2

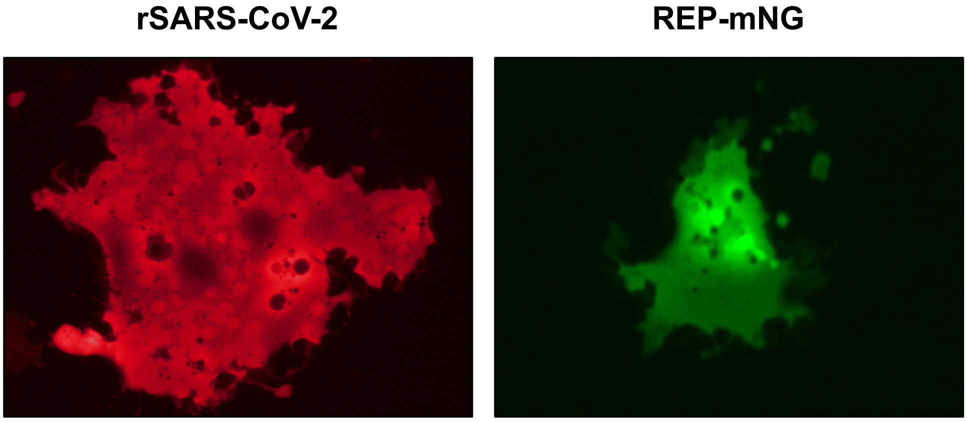

Supplementary Figure 2. Syncytia formation by SARS-CoV-2 infected cells. VeroE6-TMPRSS2 cells were either infected with rSARS-CoV-2 virus at a moi of 0.1 or transfected with SARS-CoV-2 replicon (REP-mNG). At 24 hpi cells were fixed and infected cells were detected with a polyclonal antibody specific for N protein and a secondary antibody conjugated with AlexaFluor594 (red, left panel). At 48 hpt, NeonGreen fluorescence was analyzed in transfected cells (green, right panel). Representative images of the observed syncytia are shown.
