# Supplementary material for: Cell type dependent stability and virulence of a recombinant SARS-CoV-2, and engineering of a propagation deficient RNA replicon to analyze virus RNA synthesis
Source: Front Cell Infect Microbiol. 2023 Oct 24;13:1268227. doi: 10.3389/fcimb.2023.1268227 (PMC10628495; doi:10.3389/fcimb.2023.1268227)
Supplement: Supplementary file 2 [file Table_1.docx]

**Supplementary Table 1. Oligonucleotides for SARS-CoV-2 used for sequencing.** Designed from SARS-CoV-2 Wuhan-Hu-1 isolate (GenBank MN908947)

| **Oligonucleotide** | **Sequence 5’-3’** | **Position in viral genome** |
| --- | --- | --- |
| WH_15_VS | CCTTCCCAGGTAACAAACC | 15-33 |
| WH_770_VS | GTTACCCGTGAACTCATGC | 770-788 |
| WH_785_RS | TGAGTTCACGGGTAACACC | 767-785 |
| WH_1594_VS | CGAAGGTCTTAATGACAACC | 1,594-1,613 |
| WH_1613_RS | GGTTGTCATTAAGACCTTCG | 1,594-1,613 |
| WH_2433_VS | CCAGAGAAGAAACTGGCC | 2,433-2,450 |
| WH_2450_RS | GGCCAGTTTCTTCTCTGG | 2,433-2,450 |
| WH_3233_VS | CAACAAGACGGCAGTGAGG | 3,233-3,251 |
| WH_3248_RS | CACTGCCGTCTTGTTGACC | 3,230-3,248 |
| WH_4058_VS | CCAGATTCTGCCACTCTTG | 4,058-4,076 |
| WH_4073_RS | GAGTGGCAGAATCTGGATG | 4,055-4,073 |
| WH_4800_VS | AAGATTGGTCCTATTCTGG | 4,800-4,818 |
| WH_4824_RS | GATTGTCCAGAATAGGACC | 4,806-4,824 |
| WH_5616_VS | AGATACCTTGTACGTGTGG | 5,616-5,634 |
| WH_5627_RS | TACAAGGTATCTGAACACC | 5,609-5,627 |
| WH_6467_VS | ACTACCGAAGTTGTAGGAG | 6,467-6,485 |
| WH_6489_RS | ATGTCTCCTACAACTTCGG | 6,471-6,489 |
| WH_7281_VS | ATGTACTTGGATTGGCTGC | 7,281-7,299 |
| WH_7307_RS | GCATGATTGCAGCCAATCC | 7,289-7,307 |
| WH_8105_VS | GCAACTGCAGAAGCTGAAC | 8,105-8,123 |
| WH_8123_RS | GTTCAGCTTCTGCAGTTGC | 8,105-8,123 |
| WH_8943_VS | GTAACATCTGTTACACACC | 8,943-8,961 |
| WH_8960_RS | GTGTGTAACAGATGTTACC | 8,942-8,960 |
| WH_9728_VS | TGGTTCTTTAGTAATTACC | 9,728-9,746 |
| WH_9747_RS | AGGTAATTACTAAAGAACC | 9,729-9,747 |
| WH_10543_VS | CCATATGGAATTACCAACTGG | 10,543-10,563 |
| WH_10563_RS | CCAGTTGGTAATTCCATATGG | 10,543-10,563 |
| WH_11369_VS | GATGATGGTGCTAGGAGAG | 11,369-11,387 |
| WH_11396_RS | GTGTCCACACTCTCCTAGC | 11,378-11,396 |
| WH_12159_VS | AGCAGGCTGTTGCTAATGG | 12,159-12,177 |
| WH_12178_RS | ACCATTAGCAACAGCCTGC | 12,160-12,178 |
| WH_12987_VS | TGGTACTTGGTAGTTTAGC | 12,987-13,005 |
| WH_13001_RS | AACTACCAAGTACCATACC | 12,983-13,001 |
| WH_13757_VS | TAGACGGTGACATGGTACC | 13,757-13,775 |
| WH_13779_RS | ATGTGGTACCATGTCACCG | 13,761-13,779 |
| WH_14554_VS | CTTGTGTATGCTGCTGACC | 14,554-14,572 |
| WH_14572_RS | GGTCAGCAGCATACACAAG | 14,554-14,572 |
| WH_15373_VS | TGTTGTAGCTTGTCACACC | 15,373-15,391 |
| WH_15389_RS | TGTGACAAGCTACAACACG | 15,371-15,389 |
| WH_16202_VS | ATGAGGCTATGTACACACC | 16,202-16,220 |
| WH_16225_RS | TATGCGGTGTGTACATAGC | 16,207-16,225 |
| WH_17066_VS | ATTCTACACTCCAGGGACC | 17,066-17,084 |
| WH_17098_RS | TACCAGTACCAGGTGGTCC | 17,080-17,098 |
| WH_17860_VS | GACTATGTCATATTCACTC | 17,860-17,878 |
| WH_17878_RS | GAGTGAATATGACATAGTC | 17,860-17,878 |
| WH_18702_VS | AGACACTTATGCCTGTTGG | 18,702-18,720 |
| WH_18715_RS | AGGCATAAGTGTCTGAAGC | 18,697-18,715 |
| WH_19548_VS | AGCTGGCTTTAGCTTGTGG | 19,548-19,566 |
| WH_19565_RS | CACAAGCTAAAGCCAGCTG | 19,547-19,565 |
| WH_20342_VS | GTCATAGTCAGTTAGGTGG | 20,342-20,360 |
| WH_20360_RS | CCACCTAACTGACTATGAC | 20,342-20,360 |
| WH_21142_VS | GCTCTTGGAGGTTCCGTGG | 21,142-21,160 |
| WH_21160_RS | CCACGGAACCTCCAAGAGC | 21,142-21,160 |
| WH_21923_VS | AATAACGCTACTAATGTTG | 21,923-21,941 |
| WH_21938_RS | CATTAGTAGCGTTATTAAC | 21,920-21,938 |
| WH_22763_VS | GTAATTAGAGGTGATGAAG | 22,763-22,781 |
| WH_22781_RS | CTTCATCACCTCTAATTAC | 22,763-22,781 |
| WH_23538_VS | CATATGAGTGTGACATACC | 23,538-23,556 |
| WH_23574_RS | CATATACCTGCACCAATGG | 23,556-23,574 |
| WH_24381_VS | CCACAGCAAGTGCACTTGG | 24,381-34,399 |
| WH_24399_RS | CCAAGTGCACTTGCTGTGG | 24,381-34,399 |
| WH_25155_VS | TCGATCTCCAAGAACTTGG | 25,155-25,173 |
| WH_25174_RS | TCCAAGTTCTTGGAGATCG | 25,156-25,174 |
| WH_25907_VS | GTGATGGCACAACAAGTCC | 25,907-25,925 |
| WH_25924_RS | GACTTGTTGTGCCATCACC | 25,906-25,924 |
| WH_26678_VS | TTTCCTCTGGCTGTTATGG | 26,678-26,696 |
| WH_26699_RS | TGGCCATAACAGCCAGAGG | 26,681-26,699 |
| WH_27452_VS | ACCAAGAGTGTGTTAGAGG | 27,452-27,470 |
| WH_27471_RS | ACCTCTAACACACTCTTGG | 27,453-27,471 |
| WH_28177_VS | TGGGTAGTCTTGTAGTGCG | 28,177-28,195 |
| WH_28196_RS | ACGCACTACAAGACTACCC | 28,178-28,196 |
| WH_28943_VS | CTTGACAGATTGAACCAGC | 28,943-28,961 |
| WH_28957_RS | GTTCAATCTGTCAAGCAGC | 28,939-28,957 |
| WH_29610_VS | TGTGCAGAATGAATTCTCG | 29,610-29,628 |
| WH_29629_RS | ACGAGAATTCATTCTGCAC | 29,611-29,629 |
